# simKAP: simulation framework for the kidney allocation process with decision making model

**DOI:** 10.1038/s41598-023-41162-w

**Published:** 2023-09-29

**Authors:** Yunwei Zhang, Anne Hu, Yingxin Lin, Yue Cao, Samuel Muller, Germaine Wong, Jean Yee Hwa Yang

**Affiliations:** 1 School of Mathematics and Statistics, The University of Sydney, F07- Carslaw Building, Sydney, NSW Australia; 2 Charles Perkins Centre, The University of Sydney, Sydney, NSW Australia; 3 Sydney Law School, The University of Sydney, Sydney, NSW Australia; 4 School of Mathematical and Physical Sciences, Macquarie University, Sydney, NSW Australia; 5 Sydney School of Public Health, The University of Sydney, Sydney, NSW Australia; 6 Centre for Kidney Research, Kids Research Institute, The Children’s Hospital at Westmead, Sydney, NSW Australia; 7 Centre for Transplant and Renal Research, Westmead Hospital, Sydney, NSW Australia

**Keywords:** Kidney diseases, Health care, Scientific data, Statistics

## Abstract

Organ shortage is a major barrier in transplantation and rules guarding organ allocation decisions should be robust, transparent, ethical and fair. Whilst numerous allocation strategies have been proposed, it is often unrealistic to evaluate all of them in real-life settings. Hence, the capability of conducting simulations prior to deployment is important. Here, we developed a kidney allocation simulation framework (simKAP) that aims to evaluate the allocation process and the complex clinical decision-making process of organ acceptance in kidney transplantation. Our findings have shown that incorporation of both the clinical decision-making and a dynamic wait-listing process resulted in the best agreement between the actual and simulated data in almost all scenarios. Additionally, several hypothetical risk-based allocation strategies were generated, and we found that these strategies improved recipients’ long-term post-transplant patient survival and reduced wait time for transplantation. The importance of simKAP lies in its ability for policymakers in any transplant community to evaluate any proposed allocation algorithm using in-silico simulation.

## Introduction

Organ transplantation saves lives and improves the quality of life of patients with end-organ failure^1^. However, donor organ shortage is a major impediment to successful transplantation and is an on-going and universal problem globally^2^. Currently, in Australia, there are over 10,000 patients with kidney failure requiring kidney transplantation on the waiting list, but less than 50% receive a deceased donor organ annually^3^. In the United States (US), the number of patients on the deceased donor organ list has doubled over the past decade, reaching over 150,000 patients and approximately 10% of these patients on the waiting list die while waiting for a donor kidney^4^.

Deceased donor kidneys are considered as national and community resources^5, 6^. Therefore, the decisions to allocate this scarce resource must consider not only the efficient use of organs to maximize health outcomes but also the equitable use, that is considering societal factors to address the potential imbalance. In recent years, concerted efforts have been made by the global transplant community to optimize the utilization of deceased donor organs and improve the equity in access for disadvantaged populations^7, 8^. Internationally, many countries, including the US and countries in Europe, have adopted longevity-matching (or risk-based) strategies that aim to maximize life years and quality adjusted life years by matching the quality of the donors with the projected life expectancy of the recipients^9, 10^. Any proposed changes to the allocation rule are likely to be the subject of considerable debate, because alterations to the allocation algorithm in the context of limited pool of deceased donor organs, will inevitably prioritize transplantation access to certain groups of individuals. Thus, precludes the allocation of these precious resources to others who are also equally deserving. Currently many of the existing allocation models lack evidence to support that they will perform effectively and efficiently under realistic departures from the original assumptions and data for which the algorithms were trained and tested on. Therefore, modelling the potential impact of considered changes to allocation rules based on local factors using an objective evaluation framework prior to implementation will guide adaptation, deployment and applicability in real-life settings.

There are a limited number of simulation frameworks for allocation algorithms in the literature, with the most well-established model being the Simulated Allocation Model (SAM)^11^, which was initially developed for liver transplantation but was later expanded to include kidney and pancreatic transplantation (SRTR). In addition to SAM, several other countries including the Netherlands based on the Eurotransplant system^12^, and Spain^13^, have also developed similar algorithms specifically to capture the allocation process in their country. Most of these simulation frameworks are not adaptable to the input of another proposed allocation rule and it is focused on a specific allocation system only. Other work in simulation consists of post allocation acceptance process^14, 15^, models with a special focus on candidate list generation^16, 17^ and algorithms^18^ that primarily simulate the actual allocation rules. However, these methodologies do not include mechanisms for capturing the increasingly prevalent use of shared decision-making in donor-kidney acceptance.

In this study, using deceased donor kidney transplantation as an example, we developed a flexible simulation framework for the Kidney Allocation Process (simKAP) that considers both the dynamic changes of the candidate waiting lists and a joint decision-making process between the candidates and clinicians. This framework is not organ specific and therefore, it is also generalizable to other non-kidney related allocation challenges. In this study, we evaluated the performance of our simulation model using data from the Australian and New Zealand Dialysis and Transplant (ANZDATA) Registry, demonstrating that simKAP better reflects the reality and as such, offers the opportunity to deepen the understanding of different kidney allocation processes. We further illustrated the potential of simKAP to guide policies that aimed to mitigate inherent allocation bias and adhere to agreed societal values.

## Methods

### Dataset

Data from the ANZDATA registry was used for the modelling of simKAP. This data consists of all Australian candidates (*n* = 7740) on the waiting list who received a matched deceased donor kidney with starting replacement therapy time (KRT) between 30th June 2006 and 13th November 2017. Multiple kidney transplantations were included, but multi-organ transplantations were excluded. The deceased donor waiting list data was obtained from the ANZDATA 40th Annual report by the National Organ Matching System (NOMS)^19^.

### Simulation of Kidney Allocation Process (simKAP)

**Figure 1:**
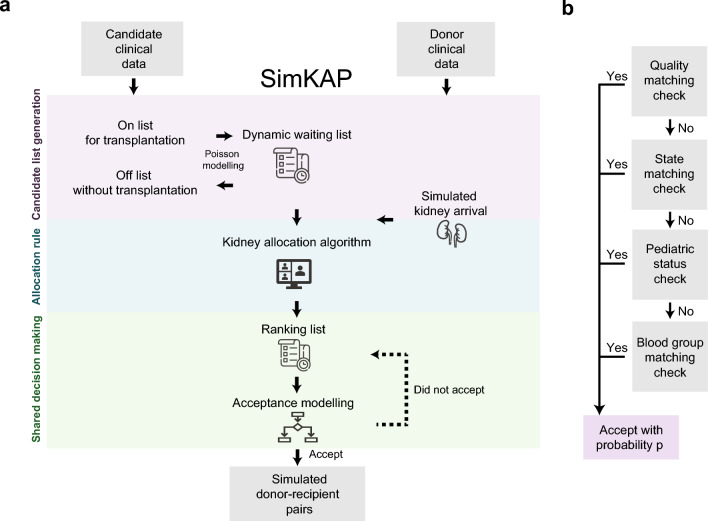
SimKAP Schematic of our simulation model. (**a**) simKAP workflow. simKAP takes kidney transplant candidate and donor data as inputs and outputs the new simulated recipient-donor pairs. Three phases implemented in simKAP are illustrated from the top panel to the bottom panel: Candidate list generation; Allocation rule; Shared decision making. (**b**) Decision making algorithm based on the sdm1 model.

The simKAP framework consists of three stages: (A) generation of the candidate list, (B) creation of the allocation rule, and (C) development of a shared-decision-making. The simKAP framework is flexible and allows for the inclusion of other allocation algorithms as an alternative; we illustrate this by providing two hypothetical allocation algorithms. The simulation requires two specific data inputs (Fig. 1): first, candidate clinical information, and second, donor clinical information. The simulated donors were first generated by selecting a random sample of simulated donors (default size of random sample is 800) and sorted chronologically according to the transplant date. The simulation procedure simKAP then sequentially took each simulated donor individually to generate a corresponding simulated recipient.

### Phase A: Candidate list generation

Based on a given candidate clinical data, the simulation procedure, simKAP first generated 300 initial candidates denoted by the set *W*_0_ = {*w*_1_,…,*w*_300_} and the notation *W*_*t*_ represents the set of individuals that were on the waiting list at time point *t*. The initial number of candidates on the waiting list were chosen such that the ratio in simKAP was similar to the ratio of the annual number of disease donors (DD graft) and the annual number of candidates “made active” and “taken off the list” from the Australian Transplant waiting list between 2011 and 2016 (Table 6.1 of the ANZDATA 40th Annual Report 2017)^19^. Next, a stratified sampling strategy was implemented for the selection of appropriate candidates for waiting listing. Under this stratified sampling strategy, weights were selected such that the simulated waiting list distribution reflected the actual waiting list distributions. The four individual characteristics taken into consideration were recipient age, blood groups, residing states of the candidates and sensitization levels. These variables were selected based on clinical knowledge that these are known risk factors for post-transplant allograft and patient outcomes.

The arrival to the candidate waiting list and exit from the candidate waiting list without transplant were modelled using Poisson processes. Here, a Poisson process queuing model with the Poisson rate parameter λ_in_ (default λ_in_ = 0.7) representing the daily arrival rate of waiting listed candidates was used to simulate the number of incoming recipients and their corresponding simulated waiting list start date. Next, a Poisson process queuing model, with the Poisson rate parameter λ_out_ (default λ_out_ = 0.4) representing the daily departure rate, was used to model the number of potential candidates being put on the interim list for a number of reasons including acute illnesses and hospitalization. To select the designated recipients leaving the waiting list without a transplant, we first estimated likelihood of temporary departure from the waiting list by calculating the probability of “exit without transplant” for all individuals currently on a waiting list using a Random Survival Forest (RSF) model with eleven recipient features, including recipient age, gender, lung disease, smoking status, diabetes, cardiovascular disease, and cancer condition. Then, *n*_out_, the number of recipients exited from the list without a transplant, was obtained based on a Poisson model with mean λ_out_. The *n*_out_ individuals with the highest exit risk score were then removed from the current waiting list *W*_*t*_. In addition, at a given time *t*, a dynamic rate parameter *k*_in_(*t*)λ_in_ and *k*_out_(*t*)λ_out_ instead of λ_in_ and λ_out_, respectively, was used in the model. This allowed for varying rates across different time periods and captured the yearly fluxes or trends in daily arrival or departure rates.

### Phase B: Allocation rule

We implemented the Kidney allocation in Australia as a default allocation rule and another two hypothetical allocation rules in the current simKAP implementation.

Australia National and State allocation algorithm in Australia is a two-tiered system. All individuals with allocation scores greater than 54,000,000 are allocated interstate under the national-based system. If no match is found, the remaining candidates are allocated under the state-based system, which occurs within the ‘home’ state where the donation occurs. TSANZ guidelines^20^ define the national allocation score as well as the five state (New South Wales (NSW), Australia Capital Territory (ACT), Victoria (VIC), Tasmania (TAS), Western Australia (WA), South Australia(SA), Northern Territory (NT), Queensland(QLD)) allocation score algorithms (NSW/ACT, VIC/TAC, WA, SA/NT and QLD) (detailed codes are available on Github at https://github.com/SydneyBioX/simKAP). For state-based allocation, recipients and donors are allocated locally. ACT, TAS and NT do not possess an allocation centre and are allocated in their respective geodesic allocation centres, that is to NSW, VIC and SA, respectively. In addition, the simKAP allocation algorithm framework implemented the following special situations:

- National-state-national allocation rounds: where the first national-state round follows the ABO-strict match for blood group, and if the donor is not matched, the second national round will attempt to match a donor-recipient pair under a more general ABO-compatible match (Table 1).
- State balance mechanism: Based on Level 7 of the national allocation algorithm in Online Appendix C of the *Clinical Guidelines for Organ Transplantation from deceased Donors*, simKAP provides an option to minimize state imbalance and maintains similarity between the number of donors to transplants within each state; that is, for a given state, the ratio between number of donors and the number of transplants is close to one on a yearly basis. For those states with this ratio greater than one, the sequential donated kidneys are allocated to a recipient in a state, experiencing a previous deficit of kidneys, based on the allocation score.

**Table 1:** Blood group compatibility match rule illustrates possible non-strict match.

		Donor blood group			
		O	A	B	AB
Recipient blood group	O	Yes	No	No	No
	A	Yes	Yes	No	No
	B	Yes	No	Yes	No
	AB	Yes	Yes	Yes	Yes

### Phase C: Shared decision-making

In the current simKAP package, a corresponding list of allocation scores of the candidate list was matched to every incoming donor kidney through a shared decision-making process and created the final donor-recipient match (or whether to accept or reject a proposed match). The four “consideration variables” that were included in this framework for the *j*th individual are:

- Pediatric status: *α*_1*j*_ = I(age_*j*_ < 18 and |age_*j*_—age_donnor_|< 30), where I(•) denotes the indicator function which has value one if the condition is satisfied and is zero otherwise;
- Donor and recipient quality measured by kidney donor risk index (KDRI) and recipient estimated post-transplant survival (EPTS): *α*_2*j*_ = EPTS_*j*_*−*KDRI_donor_;
- For recipients having blood type AB, they are more likely to accept a kidney from the same blood type.: *α*_3*j*_ = I(ABO_*j*_ = AB and ABO_donor_ = AB); and
- Donor and recipient state to account for practical benefits of not needing to transport kidneys across interstate borders: *α*_4*j*_ = I(State_*j*_ = State_donor_).

Using the simKAP framework, we implemented two shared decision-making models (sdm0 and sdm1) to capture clinician-patient decision-making in the donor-recipient matching process.

The first (sdm0) is a matching process which selects the donor-recipient pair with the highest allocation score.

The second (sdm1) is based on a nested decision tree process (Fig. 1b). We first re-order all the eligible candidates by descending order based on allocation scores calculated in Part B of simKAP. We then attempted to match a given *j* individual with the donor under *α*_*ij*_, *i* = 1, 2, 3, or 4. When attempts under all four considerations had been exhausted, and a donor-recipient match was not found, simKAP then sought to match the (*j* + 1)st individual with the donor.

The acceptance probabilities for all four considerations were modelled by a Bernoulli distribution with acceptance probability *p*_*j*_, which differs according to different recipient’s characteristics (PRA and HLA). For a typical recipient *j*, the weighted human leukocyte antigen (HLA) mismatch is defined as HLA_*j*_ = 2HLA_DR*j*_ + HLA_A_*j*_ + HLA_B_*j*_, where HLA_DR_*j*_, HLA_A_*j*_ and HLA_B_*j*_ represent recipient’s *j* mismatch counts from HLA-DR, HLA-A and HLA-B, respectively. This weighted sum aims to capture the different importance the allocation community currently places on HLA-DR versus HLA-A and HLA-B, respectively. This aspect can be customized. The PRA score is a percentage value between 0 and 100 capturing the sensitivity of an individual to HLA antigens. We use a transformed value (1−PRA_*j*_/100), such that a value close to 0 represents an individual with high sensitivity. The acceptance probability *p*_*j*_ takes the product of two transformed scores and is a value between 0 and 1. This is written as *p*_*j*_ = (1−PRA_*j*_/100)(1−HLA_*j*_/*z*), where *z* is a tuning parameter allowing users to place different emphasis between sensitivity and HLA mismatch. Our current default choice for *z* is 50. An individual with high sensitivity (small (1-PRA_*j*_/100) value) and high mismatch (small (1-HLA_*j*_/*z*)) is less likely to accept.

### Alternative allocation algorithms

We then implemented the two alternatives of risk-based allocation algorithms (B1 and B2) as detailed in Table 2. Both algorithms applied risk-based matching system that involved preferential matching of the recipient and donor pair based on the Kidney Donor Profile Index (KDPI), a measure of the donor kidney quality (the lower KDPI the better the quality) with the EPTS measuring the recipient health condition (the lower EPTS the better the health condition). The EPTS and the KDRI scores have both been externally validated in the Australian kidney transplant populations^21^. These scores are moderately good at discriminating post-transplant survival of adult kidney transplant recipients as well as allograft survivals.

**Table 2:** Hypothetical allocation algorithms and their corresponding compositions in terms of eligibility (A), allocation rule (B) and decision making (C).

	Name	Function name	A: Eligibility	B: Allocation rule	C: Decision Making
1	NationalCurrent	selection_default	*Default with dynamic waiting list*	B0: National	sdm0 approach
2	CoRisk_20	selection_corisk		B1 with $c = 20\%$	
3	CoRisk_40	selection_corisk		B1 with $c = 40\%$	
4	IRisk_20	selection_irisk		B2 with $s = 20\%$	
5	IRisk_40	selection_irisk		B2 with $s = 40\%$	

[B1] Cut-off Risk based allocation process (CORisk allocation). For a given risk-cut-off number $c$, the candidates with EPTS scores of *c*% or less were offered kidneys from donors with KDPI scores of % or less and the candidates with EPTS scores of % or more received offers from donors with KDPI scores of *c*% or more. Our candidate EPTS values were recalculated as

$$\begin{aligned} Raw\;EPTS = & 0.049 \times \left( {Recipient\;age} \right) \times I\left( {Recipient\;age\; > \;25} \right) + 0.493 \times \left( {Prior\;kidney\;transplant} \right) \\ & + 0.287 \times \log \left( {Years\;on\;dialysis + 1} \right) + 0.598 \times \left( {``Years\;on\;dialysis\; = 0^{\prime\prime}} \right), \\ \end{aligned}$$

where I(“Recipient age > 25”) and I(“Years on dialysis = 0”) are both indicator functions. The CORisk was implemented in the function ‘selection_corisk’.

[B2] Interval Risk based allocation process (IRisk allocation). For a given risk-cut-off number $s$, the candidates with EPTS scores within a given bandwidth received offers based on the KDPI scores.. The EPTS bandwidth for a given KDPI score was defined as the interval between (KDPI-*s*%) and (KDPI + *s*%). IRisk was implemented in the function ‘selection_irisk’.

The implementation of simKAP is made available in the statistics software R through a package available at https://github.com/SydneyBioX/simKAP. The package allows for the simulation of multiple scenarios through modification of each of the function arguments of simKAP. User details can be found in the package vignette.

### Performance validation

Three processes were compared in this performance evaluation:

- Process I: Maximum score selection without dynamic waiting list and shared decision- making (Phase B);
- Process II: Shared decision-making without dynamic waiting list (Phases B and C);
- Process III: Both shared decision-making and dynamic waiting list (Phases A, B and C).

We have developed an “Allocation Characteristics Comparison Analysis” (ACCA) workflow to comprehensively validate the three processes of simKAP based on allocation data characteristics between the actual and simulation results for the following allocation characteristics:

1. Ratio of National-to-State based allocations.
2. Recipient waiting time.
3. Percentages of donor kidneys allocated under different States.

For each of the above-mentioned quantities, we used the metrics listed below:

- The difference between the two values.
- The median difference between the two values after stratification.
- The Hellinger distance between the two values, which calculates the distance between two categorical vectors. The smaller the Hellinger distance the closer the characteristics.
- Kolmogorov–Smirnov (KS) statistic and the kurtosis statistic between the two values, which quantify the similarity between two distributions. The smaller these values, the more similar the two distributions.

### Ethical approval and informed consent

There is no ethics requirement for this as this is a de-identified registry dataset.

## Results

### simKAP presents a simulation framework for the Kidney allocation process

Figure 1a shows the simKAP, a flexible and dynamic simulation framework, that incorporates a shared decision-making process for deceased donor kidney allocation. The three stages are (A) generating potential transplant candidates, (B) rule and definition for computing a utility-based allocation score, and (C) selecting donor-recipient couples through decision-making. In part A, simKAP generated a list of transplant candidates at a specific time using the Poisson processes to dynamically model the arrival and departure of model candidates. In part B, the allocation algorithms (current or proposed) automatically granted an allocation score for all transplant candidates with a given simulated donor. In part C, a shared decision-making process was evaluated and observed resulting in a selected recipient for a particular donor.

A shared decision-making process is important to both healthcare professionals and patients when discussing the concepts of kidney allocations and transplantation, since there are many different options, competing concerns and risks that are unique to transplant recipients^22^. Such a decision requires a partnership between health professionals and patients with action required from all parties. For example, the factors that patients and health professionals prioritize in accepting or declining a deceased donor kidney offer are likely to be different. Prior study has reported patients placed the greatest value on kidney quality and predictors of transplant outcome^23^. Therefore, we have included donor and recipient quality measured by KDRI and EPTS along with three other variables to capture clinician-patient decision-making in the donor-recipient matching process (see Methods). Together, simKAP was built with the capacity of replicating and reflecting real-life allocation processes. Users can customize the individual components within the simKAP package.

### simKAP’s architecture reflects observed national-to-state allocation proportions

**Figure 2:**
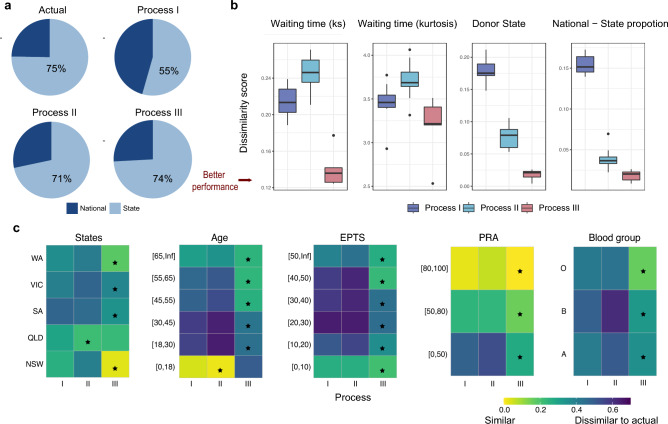
Comparison of different allocation simulation processes. (**a**) Comparison of kidney allocated proportions via the national and the state algorithm. (top left: Actual proportion, top right: Process I, bottom left: Process II, bottom right: Process III) (**b**) Dissimilarity scores for allocation characteristics, smaller scores indicates better performance of simulation (left to right: recipient waiting time, donor state and national-state proportion) (**c**) Heatmap of median recipient waiting time |log(actual/simulated)| for different recipient groups (left to right: States, Age, EPTS, PRA, Blood group); a small value close to 0 indicates good similarity between actual and simulated values. Black stars indicate the best process in each category.

To establish the most realistic allocation procedure, we compared three different allocation processes that captured the various combinations of the three parts of the allocation processes using the independent validation dataset, as detailed in Fig. 2a. We anticipated that the realistic allocation procedure in a two-tier allocation system would reflect the observed proportion allocated under the national scheme. In Australia, approximately 25% and 75% of deceased donor kidney transplants were allocated using the national and state allocation schemes, respectively. The process I represents a simulation algorithm that focuses solely on the rules and definition assigned by the allocation system (Phase B), Fig. 2a (top-right panel) shows a (45:55) national-state allocation ratio which does not reflect the actual value (25:75; Fig. 2a top-left panel). We then examined another two processes that incorporated shared decision-making (Process II) and all three phases (Process III) in the allocation process which show improved performances.

### Comprehensive assessment demonstrates simKAP’s capacity to capture actual results

We then validated our predictions against the actual results and compared several allocation characteristics through the calculation of a dissimilarity score between simulated and actual results characteristics (see Method, Performance validation). Overall, Process III had the best performance as illustrated by having the smallest dissimilarity score (Fig. 2b) across recipient waiting time, donor state and national-state proportions. Moreover, the summarized dissimilarity score decreased from 0.3 to 0.15 when comparing results from Process III and Process I. Figure 2c further illustrates the superior performance of Process III with the stratification of the median waiting time by categories of recipient characteristics and included recipient EPTS, recipient PRA level, recipient age groups, recipient blood group and the donor state. We observed Process III had the smallest values (the best) for all (20 out of 21) except for pediatric recipients (age < 18) group.

### Simulation modelling of alternative allocation models highlights that risk-matching improves recipients long-term post-transplant survival

**Figure 3:**
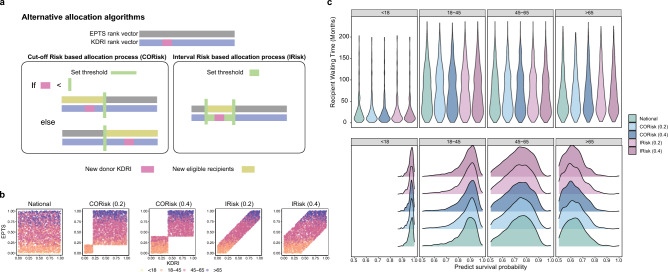
(**a**) Schematics of alternative allocation algorithms: left, Cut-off risk based allocation process (CORisk) algorithm, right: Interval Risk based allocation process. (**b**) Justification of different allocation algorithms (National, CORisk (0.2), CORisk (0.4), IRisk (0.2), IRisk (0.4), see Table 2): x-axis indicates KDRI scores, y-axis indicates EPTS scores, color by recipient age group. (**c**) Comparison of major allocation characteristics in terms of predicted survival probability under different allocation algorithms: top: recipient waiting time (months), bottom: survival probability.

To illustrate simKAP’s potential to inform policy makers in allocation algorithms, we simulated additional hypothetical allocation algorithms and observed the impact on how the deceased donor kidney was allocated among the different transplant candidates. We implemented two hypothetical types of risk-based allocation algorithms (Fig. 3a): CORisk and IRisk processes (see Method: Alternative allocation model). Both risk-based allocation procedures were inspired by the current algorithm for US new KAS Kidney transplant allocation system^24^.

Next, we examined the simulated results of five different allocation processes detailed in Table 2. Figure 3b shows the effects of the risk-based allocation procedure, where the KDRI and EPTS values for each simulated donor-pair are displayed in each scatter plot. The existing method, as predicted, does not explicitly account for donor or beneficiary quality, hence no specific pattern was observed. In the remaining four scatter plots, the restriction of the various KDRI and EPTS thresholds highlighted the allocation restriction that was implemented as part of the risks-based algorithm. For example, with CORisk(0.4) model, the candidates with EPTS scores of 40% or less received offers for kidneys from donors with KDPI scores of 40% or less and the candidates with EPTS scores of 40% or more will receive offers for kidneys from donors with KDPI scores of 40% or more. The scatter plot shows that no allocation will happen between a donor with KDRI 0.5 and a candidate with EPTS 0.2.

As expected, the waiting period varied by age group, and the estimated post-transplant survival probability decreased with increasing age. In the current illustration settings, we have demonstrated (Fig. 3c) that the five different algorithms had relatively minimal influence (similar distributions) on the recipient waiting time and 10-year survival probability for candidates of various ages. Furthermore, for highly sensitive patients, the selected risk-based allocation algorithm (CORisk_0.4) provided recipients with higher quality kidneys and reduced waiting time (Supplementary Fig. 1). An overview of the comparisons between the five allocation algorithms are provided in the Supplementary Material. This demonstrates the flexibility of simKAP as a tool to examine the impact of multiple proposed allocation algorithms using in-silico simulation. Additional case studies are provided in the Supplementary Material.

## Discussion

In this novel simKAP simulation model, we used a series of allocation rules to simulate the entire process of the deceased donor kidney allocation in Australia. This model incorporated a decision-making process that takes into consideration the uncertainties of clinical decision-making in real-life settings and evaluated the downstream effects of the simulation process. Through the inclusion of the novel element of shared decision modelling, we found the simulated results from simKAP better reflect real life data.

Binary classification modelling approaches, such as logistic regression or support vector machine, have been used to predict whether a candidate will accept a given kidney or not in the literature^14^. However, the logistic regression model is dependent on specific regional information and should be built specific to each country. Here, Phase C of shared decision-making in simKAP proposes several models to capture the behavioural variabilities between clinicians and health professionals, and other models could be applied to capture behavioural variabilities in other jurisdictions, including any user-defined logistic regression models.

The choice of the starting number in the waiting list (*W*_0_), together with λ_in_ and λ_out_, will have an impact on the waiting list distribution throughout the simulation process. A higher rate of arrival and lower rate of departure will result in more transplant candidates on the waiting list. In contrast, a lower rate of arrival and higher rate of departure will result in less transplant candidates on the waiting list. Our current default setting was created such that the ratio of number of kidney grafts per year to the number of candidates arriving and departure on the waiting list was similar to the number of transplant candidates being delisted from the deceased donor waiting list. When designing the simulations for modelling the expected size of the waiting list, it is important to jointly determine the three parameters.

Computing speed is critical when it comes to establishing simulation processes, and the computation cost is typically generated by the desire for being able have many repetitions. The ability to repeat the simulation a suitable number of times to capture the variability of simulated recipient-donor pairs, contributes to the stability and reliability of the simulation findings. Hence, our simKAP model addresses the computational challenge by integrating parallel processing across many cores, thus considerably increases simulation speed. For example, resampling 20 times on a standard personal computer took 1745s (around 30 min) without parallelization whereas this reduced to 847 s (around 14 min) when we parallelized the same simulation using two cores. Our approach can distribute replications across all cores of that the device can handle concurrently rather than sequentially and thus decreases the overall computational load.

The simKAP model has many relevant clinical and policy implications. Transplant professionals are entrusted with the stewardship of deceased donor organs. Transplant clinicians are accountable for making the most appropriate decisions to ensure the donor organs are used equitably and efficiently to maximize survival gains and quality of life for those deemed suitable for transplantation. Therefore, the organ specific allocation policy should include the appropriate performance indicators, and a self-evaluation process to ensure the data and outcomes are aligned with the performance goals. The simKAP framework allows the assessment of the highly dynamic nature of the deceased donor kidney waiting list, and has the capability to evaluate these changes real-time, assuring the public that the ethical principles of organ donation and allocation are upheld, and the desired outcomes are achieved.

The current limitation of simKAP is in its capacity to handle multiple transplantation such as dual kidney-liver or kidney-pancreas transplantation. To establish such an algorithm, the simulation process can be extended to incorporate complex input that handles both the offer and acceptance of multiple organs. In addition, simKAP can be further validate in other jurisdictions. Going forward, simKAP could be repeated applied to estimate individual dynamic profile of potential transplant offers and extended to handle complex allocation scenarios which will shape future research priorities.

## Conclusion

Here we developed a simulation framework for the entire Kidney Allocation Process (simKAP) of deceased donor that incorporated a novel element of shared decision-making modelling that capture uncertainties of clinical decision-making in real-life settings. Our detailed evaluation illustrates that in many scenarios, the incorporation of both the clinical decision-making and a dynamic wait-listing process provides the best agreement between the actual and simulated data. The application of the simKAP model is broad and has the potential to influence the allocation and acceptance decisions made by the transplant health professionals and the patients.

### Supplementary Information

Supplementary Information 1.

Supplementary Information 2.

## Data Availability

For the ANZDATA, data requests can be made through the ANZDATA registry, and access to the data source will require HREC approvals. For any help required for data access, please contact GW. An R package is provided at https://github.com/SydneyBioX/simKAP to allow users to construct or customise their own models for use in other studies.

## References

[1] Wong G, Howard K, Chapman JR (2012). Comparative survival and economic benefits of deceased donor kidney transplantation and dialysis in people with varying ages and co-morbidities. PLoS ONE.

[2] Rudge C, Matesanz R, Delmonico FL (2012). International practices of organ donation. Br. J. Anaesth..

[3] Website, Reports - ANZDATA. https://www.anzdata.org.au/anzdata/publications/reports/ (2016).

[4] Danovitch GM, Michael CJ (2003). Allocation of deceased donor kidneys: past, present, and future. Am. J. Kidney Dis..

[5] Howard K, Jan S, Rose JM (2016). Preferences for policy options for deceased organ donation for transplantation. Transplantation.

[6] Meier-Kriesche H-U, Schold JD, Gaston RS (2005). Kidneys from deceased donors: Maximizing the value of a scarce resource. Am. J. Transpl..

[7] Schinstock CA, Smith BH, Montgomery RA, Jordan SC, Bentall AJ, Mai M, Khamash HA, Stegall MD (2019). Managing highly sensitized renal transplant candidates in the era of kidney paired donation and the new kidney allocation system: Is there still a role for desensitization?. Clin. Transpl..

[8] Kim Y, Ahmed E, Ascher N (2021). Meeting report: First state of the art meeting on gender disparity in kidney transplantation in the Asia-Pacific. Transplantation.

[9] Calisa V, Craig JC, Howard K (2018). Survival and quality of life impact of a risk-based allocation algorithm for deceased donor kidney transplantation. Transplantation.

[10] Lee D, Kanellis J, Mulley WR (2019). Allocation of deceased donor kidneys: A review of international practices. Nephrology.

[11] Website, *Simulated Allocation Models*. https://www.srtr.org/requesting-srtr-data/simulated-allocation-models/.

[12] Doxiadis IIN, Smits JMA, Persijn GG (2004). It takes six to boogie: Allocating cadaver kidneys in eurotransplant. Transplantation.

[13] del Río F, del Río F, Andrés A (2019). Kidney transplantation from donors after uncontrolled circulatory death: The Spanish experience. Kidney Int..

[14] Kim S-P, Gupta D, Israni AK (2015). Accept/decline decision module for the liver simulated allocation model. Health Care Manag. Sci..

[15] Aubert O, Reese PP, Audry B (2019). Disparities in acceptance of deceased donor kidneys between the united states and france and estimated effects of increased US acceptance. JAMA Intern. Med..

[16] Cechlárová K, Hančová M, Plačková D (2021). Stochastic modelling and simulation of a kidney transplant waiting list. CEJOR.

[17] Zou J, Lederer DJ, Rabinowitz D (2020). Efficiency in lung transplant allocation strategies. Ann. Appl. Stat..

[18] de Klerk M, de Klerk M, Kal-van Gestel JA (2021). Creating options for difficult-to-match kidney transplant candidates. Transplantation.

[19] Website, *ANZDATA 40th Annual Report 2017 (Data to 2016)* – (ANZDATA, 2018). https://www.anzdata.org.au/report/anzdata-40th-annual-report-2017/.

[20] Website, *TSANZ - Organ Allocation Guidelines* (Transplantation Society of Australia). https://tsanz.com.au/guidelinesethics-documents/organallocationguidelines.htm.

[21] Clayton PA, Dansie K, Sypek MP (2019). External validation of the US and UK kidney donor risk indices for deceased donor kidney transplant survival in the Australian and New Zealand population. Nephrol. Dial. Transplant..

[22] Elwyn G, Frosch D, Thomson R (2012). Shared decision making: A model for clinical practice. J. Gen. Intern. Med..

[23] Howell M, Tong A, Wong G (2012). Important outcomes for kidney transplant recipients: A nominal group and qualitative study. Am. J. Kidney Dis..

[24] Stegall MD, Stock PG, Andreoni K (2017). Why do we have the kidney allocation system we have today? A history of the 2014 kidney allocation system. Hum. Immunol..

