# Supplementary material for: simKAP: simulation framework for the kidney allocation process with decision making model
Source: Sci Rep. 2023 Sep 29;13:16367. doi: 10.1038/s41598-023-41162-w (PMC10541869; doi:10.1038/s41598-023-41162-w)
Supplement: Supplementary file 1 — Supplementary Information 1. [file 41598_2023_41162_MOESM1_ESM.docx]

**Supplementary material**

**simKAP: Simulation framework for the kidney allocation process with decision making model**

Yunwei Zhang^#1,2^, Anne Hu^#1,3^, Yingxin Lin^1,2^, Yue Cao^1,2^, Samuel Muller^1,4^, Germaine Wong*^5,6,7^, Jean Yee Hwa Yang*^1,2^

**Part A**

**Supplementary Figures:**

**
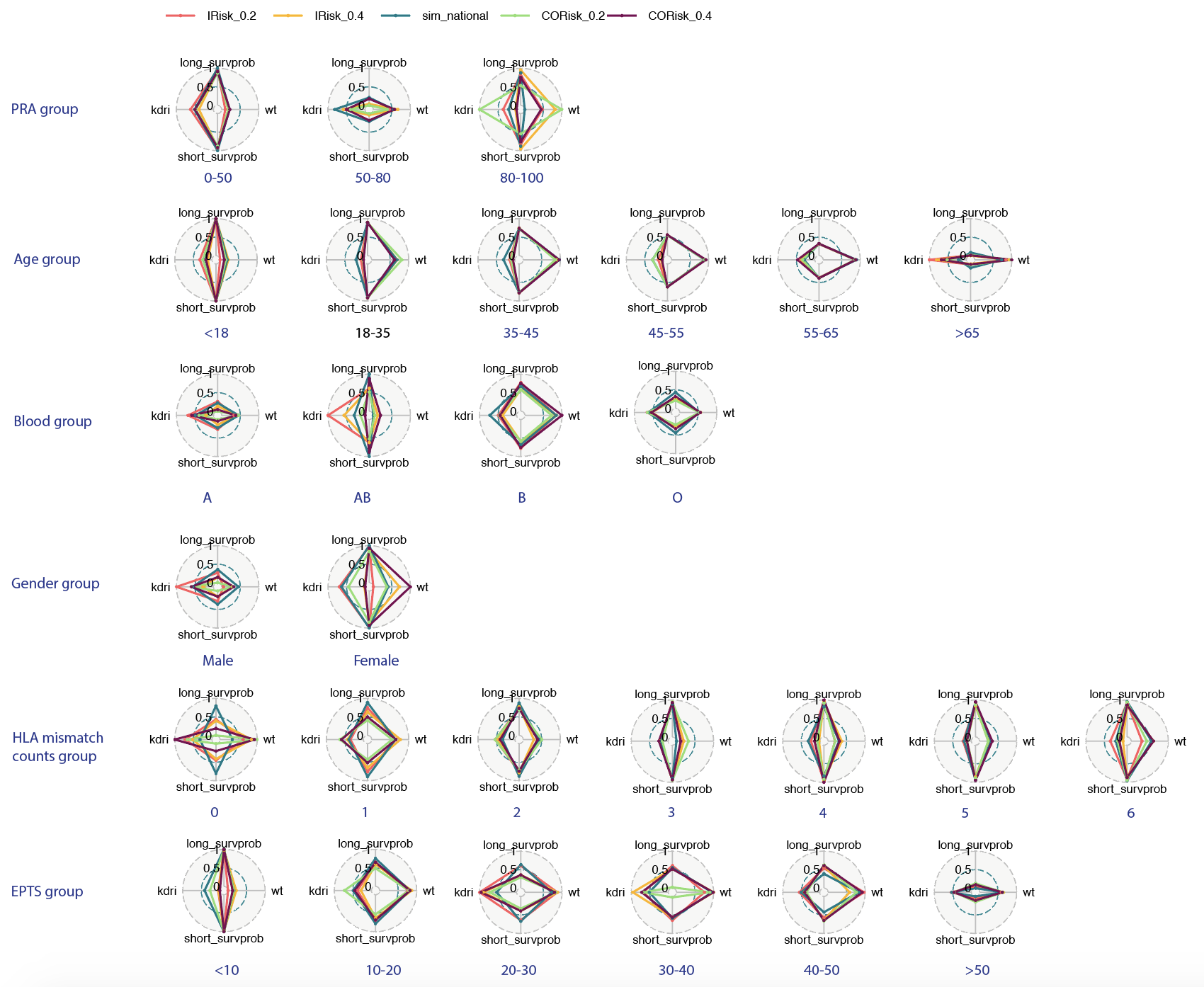
**

**Supplementary Figure1.** Radar plot for allocation algorithm evaluation.

Colors: five different allocation algorithms. Rows: recipient group stratification category. Columns: specific recipient group within each category. For each radar plot, four directions are donor kidney risk index (kdri), estimated ten-year survival probability (long_survprob), waiting time (wt) and estimated one-year survival probability (short_survprob).

**
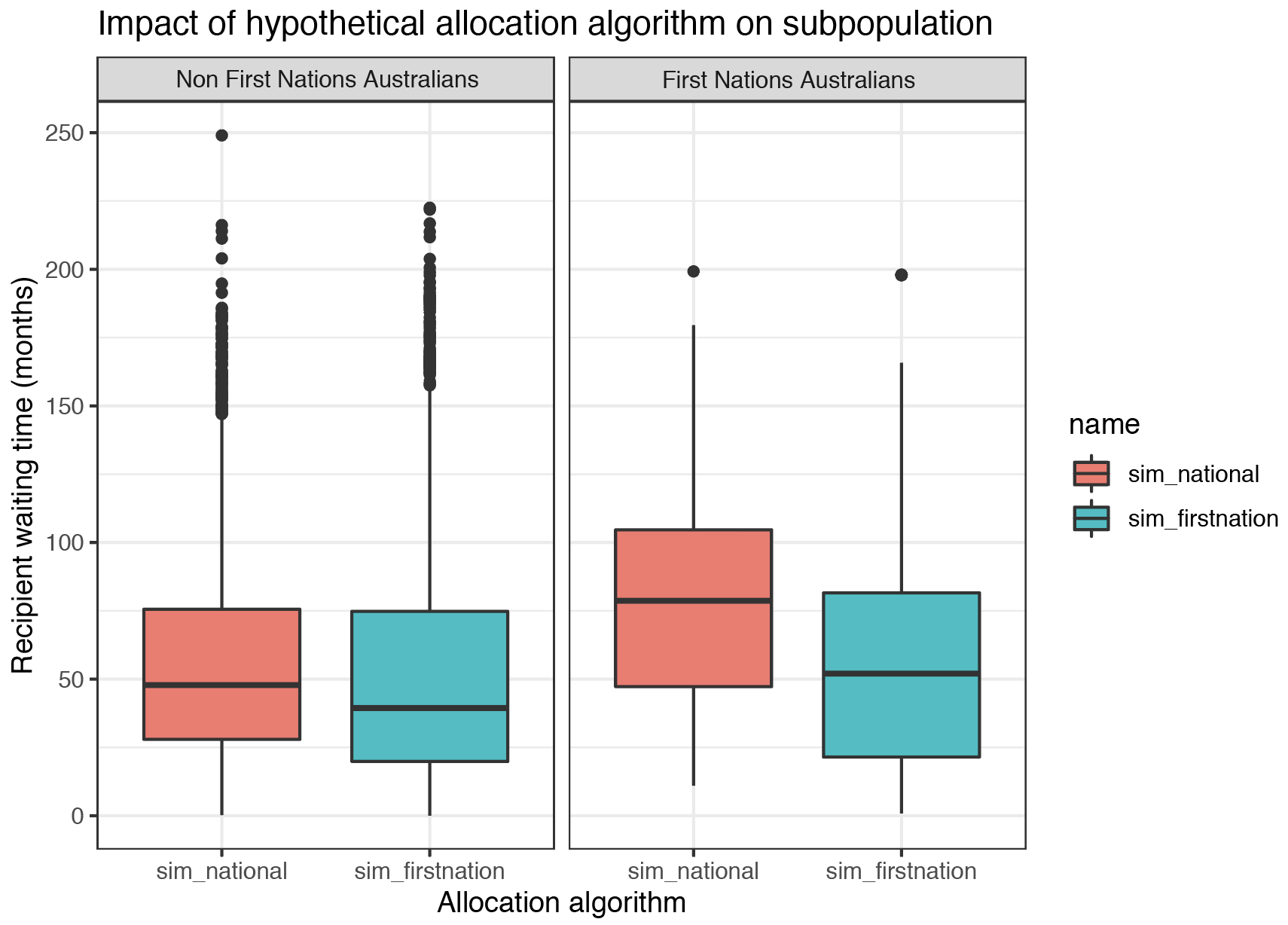
**

**Supplementary Figure2. Impact of hypothetical allocation algorithm on subpopulation.** Boxplots of waiting time (in months, y-axis) for the First Nations Australians (right) and others (left) using two different allocation algorithms. The current allocation algorithm is highlight in red (sim_national) and the hypothetical allocation algorithm including First Nations Australians bonus is highlight in Cyan (sim_firstnation).

**Supplementary Tables:**

**Supplementary Table1.** Acronyms table.

| Acronyms | Full name | Explained |
| --- | --- | --- |
| HLA | Human leukocyte antigens | They are marker proteins in the immune system to determine whether a cell belongs to your body or not. |
| HLA-A | HLA A loci | HLA-A is one of the classical HLA alleles. |
| HLA-B | HLA B loci | HLA-B is one of the classical HLA alleles. |
| HLA-C | HLA DR loci | HLA-DR is one of the classical HLA alleles. |
| EPTS | Estimated post-transplant survival | Originally developed from the US. It is used to estimate how long after a transplant surgery a patient will survive using patient’s characteristics. |
| KDRI | Kidney donor risk index | Originally developed from the US. It is used to reflect the post-transplant graft failure rate associated with the deceased donor’s kidney using donor’s characteristics. |
| PRA | Panel reactive antibody | To determine how sensitise a patient is in transplant. Usually, the more previous transplants the patient have, the higher the PRA level. |
| AOB | A,B,O,AB blood type | This is referred to the blood type that people have. |
| RSF | Random survival forest | This is a survival model used to predict survival. |
| NSW | New south wales | One state in Australia. |
| ACT | Australia Capital Territory | One state in Australia. |
| VIC | Victoria | One state in Australia. |
| TAC | Tasmania | One state in Australia. |
| WA | Western Australia | One state in Australia. |
| SA | South Australia | One state in Australia. |
| NT | Northern Territory | One state in Australia. |
| QLD | Queensland | One state in Australia. |
| DD | Deceased donor | Deceased donor. |
| ANZDATA | Australian and New Zealand Dialysis and Transplant Registry | Australian and New Zealand Dialysis and Transplant Registry |
| KRT | Replacement therapy time | A time defined. |
| NOMS | National Organ Matching System | A system. |
| TSANZ | Transplantation Society of Australia & New Zealand | A society. |
| CORisk | Cut-off Risk based allocation process | An alternative allocation rule. |
| IRisk | Interval Risk based allocation process | An alternative allocation rule. |
| ACCA | Allocation Characteristics Comparison Analysis | An evaluation workflow for the simulation process. |
| KS | Kolmogorov–Smirnov (KS) statistics | A statistics used in the evaluation workflow. |

This is the acronyms table where all acronyms are listed in the first column and the full names are in the second column followed by detailed explanations in the last column.

**Supplementary Table2.** Evaluation of allocation algorithms for different recipient groups based on allocation characteristics.

|  |  | Allocation characteristics |  |  |  |  |
| --- | --- | --- | --- | --- | --- | --- |
|  |  |  | wt | 1yr survporb | 10yr survprob | kdri |
| Recipient characteristics | Age | <18 | yes | no | no | no |
|  |  | 18-30 | no | no | no | yes |
|  |  | 30-45 | no | yes | no | yes |
|  |  | 45-55 | no | no | no | yes |
|  |  | 55-65 | no | yes | yes | yes |
|  |  | >65 | no | yes | yes | yes |
|  | EPTS | <10 | no | yes | yes | yes |
|  |  | 10-20 | no | yes | yes | yes |
|  |  | 20-30 | yes | yes | yes | yes |
|  |  | 30-40 | yes | yes | yes | yes |
|  |  | 40-50 | yes | yes | yes | yes |
|  |  | >50 | no | yes | yes | yes |
|  | Blood | A | yes | yes | yes | no |
|  |  | B | no | no | no | no |
|  |  | O | no | yes | yes | no |
|  | HLA-mismatch | 0 | no | yes | yes | no |
|  |  | 1 | no | yes | yes | yes |
|  |  | 2 | no | yes | yes | yes |
|  |  | 3 | no | no | no | no |
|  |  | 4 | no | yes | yes | no |
|  |  | 5 | no | no | no | no |
|  |  | 6 | no | no | no | no |
|  | Gender | F | no | yes | yes | no |
|  |  | M | no | yes | yes | yes |
|  | PRA | [0,50) | no | yes | yes | no |
|  |  | [50,80) | no | no | no | no |
|  |  | [80,100] | yes | no | no | no |

Each row represents the allocation characteristics: recipient wait time (wt), one-year patient survival probability(1yr survprob), ten-year patient survival probability(10yr survprob), donor KDRI (kdri). Each column represents recipient characteristics/groups. The value in the table contain “yes” and “no”. The value “yes” indicate ANOVA test shows significant difference among those 5 examined allocation algorithms and the value “no”: ANOVA test does not show significant difference among those 5 examined allocation algorithms.

**Part B**

**Supplementary results:**

**Evaluation of different allocation algorithms**

We have found that for highly sensitised patients (PRA level greater than 80, third plot in the first row), risk-based allocation algorithm, CORisk_0.4, provides recipients with higher quality kidneys (a small kdri value) and reduced waiting time (a small wt value) (Supplementary Figure 1).

For male and female patients, allocation algorithm CORisk_0.2 benefits female candidates with a higher kidney quality and a shorter waiting time without injuring male candidates with much poorer kidney qualities and longer waiting times (first and second plots in the fourth row) (Supplementary Figure 1).

**First-nation focus allocation algorithm**

We study an alternative allocation algorithm that gives preference to first national people in Australia (bonus of 1,000,000,000). Under this hypothetical allocation algorithm, we observe up to 20 months reduction in wait time for first national people aged greater than 18 and about 3 months decrease for paediatric patients compared with the current national allocation algorithm. As for other candidates, this first-national focus allocation algorithm shows comparable wait times (see Supplementary Figure 2). These results demonstrate simKAP’s ability to investigate the impact of any hypothetical algorithm on different sub-populations.
