# Supplementary material for: simKAP: simulation framework for the kidney allocation process with decision making model
Source: Sci Rep. 2023 Sep 29;13:16367. doi: 10.1038/s41598-023-41162-w (PMC10541869; doi:10.1038/s41598-023-41162-w)
Supplement: Supplementary file 2 — Supplementary Information 2. [file 41598_2023_41162_MOESM2_ESM.pdf]

# Appendix C

## Kidney allocation algorithms

### National Allocation formula

| Match level | Description | Criteria | | Base score |
|---|---|---|---|---|
| 1 | Very Highly sensitised<br>ABO Compatible | **1a** | mPRA ≥99.7 | 99 700 000 |
| | | **1b** | mPRA ≥99 | 99 000 000 |
| | | **1c** | mPRA ≥98 | 98 000 000 |
| | | **1d** | mPRA ≥97 | 97 000 000 |
| | | **1e** | mPRA ≥96 | 96 000 000 |
| | | **1f** | mPRA ≥95 | 95 000 000 |
| National Urgent | ABO Compatible | Recipient National urgency >0 | | 90 000 000 |
| 2 | EPTS restriction<br>HLA matching<br>Prioritises Low EPTS recipients<br>Matched at HLA DRB1<br>ABO Matched<br>KDPI max value is applied from this level down | **2a** | 0 mismatches HLA-A or HLA-B and EPTS ≤25 | 89 000 000 |
| | | **2b** | 1 mismatch HLA-A or HLA-B and EPTS ≤25 | 88 000 000 |
| | | **2c** | 2 mismatch HLA -A or HLA-B and EPTS ≤25 | 87 000 000 |
| | | **2d** | 0 mismatches HLA -A or HLA-B and EPTS ≤60 | 86 000 000 |
| 3 | HLA matching<br>Highly Sensitised | **3a** | 0 mismatch at HLA A or HLA B or HLA DRB1<br>and mPRA >80 | 79 000 000 |
| | | **3b** | 1 mismatch at HLA A or HLA B or HLA DRB1<br>and mPRA >80 | 78 000 000 |
| | | **3c** | 2 mismatches at HLA A or HLA B or HLA DRB1<br>and mPRA >80 | 77 000 000 |
| | HLA Matching<br>Centre credit difference | **3d** | Matched at HLA DRB1<br>1 mismatch HLA A or HLA B<br>And mPRA ≤80<br>And Centre credit difference ≤-3 | 76 000 000 |
| | | **3e** | Matched at HLA DRB1<br>2 mismatch HLA A or HLA B<br>And mPRA ≤80<br>Centre credit difference ≤-6 | 75 000 000 |
| | | **3f** | mPRA >80<br>Centre credit difference ≤-9 | 74 000 000 |
| | | **3g** | Centre credit difference <-20 | 73 000 000 |

| Other parameters | Bonus points added |
|---|---|
| **Paediatric** | 250 000 |
| **Donor centre = patient centre** | 50 |
| **Recipient Centre credit** | 1000 + recipient centre credit |
| **Recipient and Donor are HLA DRB1 homozygote** | 500 000 ( except level 3G) |
| **Waiting time (on dialysis)** | Number of months x 1 |

## State Allocation

- Allocation initially matched with restriction applied (EPTS-KDPI <=50) then unrestricted matching is applied
- KPDI max at clinician's discretion.

| Level | Description | Details | Base Score |
|---|---|---|---|
| State Urgent | State Urgency Index >0 | Urgency index added to base score | 60 000 000 |

| Level | Description | Details | Restricted base score | Unrestricted base score |
|---|---|---|---|---|
| State HLA | HLA mismatches A/B/DRB1 | **1a** 0 0 0 | 49 000 000 | 39 000 000 |
| | | **1b** 1 0 0 or 0 1 0 | 48 000 000 | 38 000 000 |
| | | **1c** 1 1 0 | 47 000 000 | 37 000 000 |
| | | **1d** 0 0 1 | 46 000 000 | 36 000 000 |
| | | **1e** 2 0 0 or 0 2 0 | 45 000 000 | 35 000 000 |
| | | **1f** 1 0 1 or 0 1 1 | 44 000 000 | 34 000 000 |
| | | **1g** 2 1 0 or 1 2 0 | 43 000 000 | 33 000 000 |
| State Waiting | Months on dialysis | Number of months x 1 | 40 000 000 | 30 000 000 |

Additional scores

- Paediatric bonus of 100 000 for restricted algorithms – state HLA and state waiting
- Recipient and donor are HLA DRB1 homozygous bonus 500 000 to state HLA matching algorithms only.

In the event that a more than one patient has the same score, the ranking is randomised.

## Interstate Utilisation Algorithm

In rare situations there may not be enough patients in a given state to be able to accept the available kidneys. Most often this occurs if the donor has a rarer blood group, such as AB. If there are not enough patients to receive the kidneys locally, a national interstate Utilisation t is run. This list incorporates patients from across the country, to ensure that the kidneys do not go to waste.

| Level | Description | Details | Restricted base score | Unrestricted base score |
|---|---|---|---|---|
| State HLA | HLA mismatches A/B/DRB1 | **1a** 0 0 0 | 19 000 000 | 9 000 000 |
| | | **1b** 1 0 0 or 0 1 0 | 18 000 000 | 8 000 000 |
| | | **1c** 1 1 0 | 17 000 000 | 7 000 000 |
| | | **1d** 0 0 1 | 16 000 000 | 6 000 000 |
| | | **1e** 2 0 0 or 0 2 0 | 15 000 000 | 5 000 000 |
| | | **1f** 1 0 1 or 0 1 1 | 14 000 000 | 4 000 000 |
| | | **1g** 2 1 0 or 1 2 0 | 13 000 000 | 3 000 000 |
| State Waiting | Months on dialysis | Number of months x 1 | 10 000 000 | 0 |

## ABO selection rules

The ABO selection rules determine the acceptable organ matches, as shown:

| Algorithm | Level | Donor ABO type | Patient ABO type |
|---|---|---|---|
| National | Level 1 | A | A |
| | | A | AB |
| | | B | B |
| | | B | AB |
| | | AB | AB |
| | | O | O |
| | | O | A |
| | | O | B |
| | | O | AB |
| National | Level 2 and Level 3 | A | A |
| | | B | B |
| | | AB | AB |
| | | O | O |
| State | NSW<br>WA<br>VIC<br>SA | A | A |
| | | A | AB |
| | | B | B |
| | | B | AB |
| | | AB | AB |
| | | O | O |
| State | QLD | A | A |
| | | A | AB |
| | | B | B |
| | | B | AB |
| | | AB | AB |
| | | O | O |
| | | O | B |
